# Postoperative complications of xenogenic dural substitutes in hemicraniectomy

**DOI:** 10.1007/s00068-026-03185-6

**Published:** 2026-04-16

**Authors:** Levent Tanrikulu, Adnan Abo Alhasan, Patrick Dömer, Johannes Woitzik

**Affiliations:** 1 Department of Neurosurgery, Carl von Ossietzky University Oldenburg, Universitätsmedizin Oldenburg, Steinweg 13-17, 26121 Oldenburg, Germany; 2 Research Center Neurosensory Science, Carl von Ossietzky University, Oldenburg, Germany

**Keywords:** Decompressive hemicraniectomy, Dural substitute, Side effects

## Abstract

**Purpose:**

Decompressive hemicraniectomy (DHC) is a treatment option for refractory intracranial pressure (ICP) elevation in cases of cerebral infarction, hemorrhage, and traumatic brain injury. Several surgical techniques are used for musculocutaneous, bony, and dural exposure. The method of dural closure, specifically whether to use expanding duraplasty with or without xenogenic dural substitutes, remains a topic of debate. This study focuses on the occurrence of postoperative surgical side effects associated with these approaches.

**Methods:**

A total of 258 patients (mean age 52.8 ± 16.7 years; 140 male, 118 female, 79 infarctions, 57 traumatic brain injuries, 55 subarachnoid hemorrhages, 32 intracranial hemorrhages, 30 subdural hematomas, 5 other indications) who underwent DHC between 2015 and 2024 at our institution were reviewed. Complete clinical and radiological data were available for all patients. The patient population was divided into two groups: • Group A: 201 patients who received the xenogenic dural substitute. • Group B: 57 patients who did not receive a dural substitute.

**Results:**

Baseline demographics (age, gender, underlying pathology) were comparable between both groups. No significant differences were found in the following parameters: • Duration of DHC surgery: 102.0 min (Group A) vs. 108.0 min (Group B); *p* = 0.24. • Postoperative CSF leakage: 3.5% (7/201) in Group A vs. 3.6% (2/55) in Group B; *p* = 0.96. Postoperative shunt dependency: 17.7% (35/198) in Group A vs. 16.4% (9/55) in Group B; *p* = 0.82. • Postoperative bleeding: 3.5% (7/198) in Group A vs. 5.5% (3/55) in Group B; *p* = 0.52. • Postoperative wound infections: 7.1% (14/198) in Group A vs. 1.8% (1/55) in Group B; *p* = 0.14. Cranioplasty was performed in 55.8% (110/197) of the patients in Group A and in 44.4% (24/54) in Group B. The use of xenogenic dural substitutes did not significantly affect the duration of cranioplasty surgery (114.2 min vs. 117.6 min; *p* = 0.72) or the cranioplasty results. However, the combined wound infection rate for both DHC surgery and cranioplasty showed a trend towards higher wound infection rates in patients who received xenogenic substitutes: • Combined wound infection rate: 11.6% (23/198) in Group A vs. 3.5% (2/57) in Group B; *p* = 0.07.

**Conclusion:**

The use of xenogenic substitutes may not significantly impact the prevention of postoperative complication profile with or without the usage of xenogenic dural substitutes in DHC. However, there was a trend towards a higher incidence of postoperative infectious complications in patients who received xenogenic dural substitutes.

## Introduction

Decompressive hemicraniectomy (DHC) represents an efficient surgical treatment option in patients with refractory intracranial hypertension after traumatic brain injury (TBI), subarachnoid hemorrhage (SAH) or imminent cerebral infarction [1, 4–7]. The principle of ICP management is approached by hemispheric bone removal and dural incision (with or without a duraplasty), enabling for sufficient expansion of the compressed brain parenchyma [3]. A craniectomy diameter of 12 cm has been postulated to represent the minimum size for effective decompression, as the incidence of hemicraniectomy-associated lesions increases sharply with defects of smaller size [8–14]. There are several techniques of durotomy and dural closures in DHC, while the surgical risk profile has not been analyzed in detail. Duraplasty is intended to protect the cortex and reduce cerebrospinal fluid (CSF) issues, it adds foreign material that might raise infection risk, and evidence for or against it. This article focuses on the impact of the use of xenogenic dural substitutes for the occurence of surgical site effects such as cerebrospinal fluid leakages, duration of surgery for DHC and subsequent cranioplasty, postoperative wound infections and postoperative hemorrhage.

## Methods

This study was approved by the local ethics committee (Medizinische Ethikkommission der Universität Oldenburg, AZ 2025-065). We retrospectively reviewed all patients undergoing DHC from 2015 to 2024 at our institution as documented in our electronical patient information system. The indication for DHC was established for the treatment of refractory ICP. The operative decompression was performed in a standard fashion with a minimum diameter of 12 cm with obligatory extension to the floor of the middle fossa. The type of duraplasty was documented in the surgical report. The decision to use a xenogenic dural graft was based on the surgeon´s preference. There were no differences in different closure choices. The interval between the index DHC and cranioplasty was three months. The types of cranioplasty was autologous in all cases and the bone flaps were stored in the abdominal subcutaneous tissue. Patients were followed up after surgery for 14 days to ascertain the occurence of an infection after DHC and cranioplasty.

A total of 258 patients who underwent DHC were analyzed. Complete clinical and radiological data were available for all patients. The patient population was divided into two groups:

- Group A: 201 patients who received the xenogenic dural substitute (Lyoplant, Aesculap, Germany, material property: bovine pericardium).
- Group B: 57 patients who did not receive a dural substitute.

In Group A xenogenic dural substitute was positioned by an onlay technique onto the dural gaps after durotomy. In Group B, the dural gaps were left open and the musculocutaneous flap was retracted back overlaying the dural gaps.

For statistical analysis the data are presented as mean ± standard deviation (SD) and as percentage. For categorial data, Chi^2^-test was used to test for statistical significance while a two-sided t-test was used for comparison of quantitative parameters. Statistical significance was set at *p* < 0.05; statistical results with *p* < 0.001 were considered as highly significant; a statistical trend was defined as 0.05 < *p* < 0.10. To control for multiple testing, Bonferroni correction was applied in the case of t-tests, resulting in a significance level of *p* < 0.025.

## Results

**Fig. 1:**
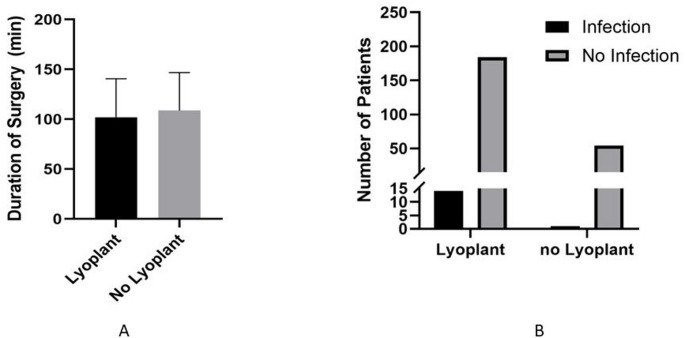
Statistical view over the duration of surgery (**A**) and infectious complications (**B**)

We were able to include 258 patients (mean age 52.8 ± 16.7 years; 140 male, 118 female) based on the availability of comprehensive clinical data sets and postoperative CT scans. DHC was performed for 79 infarctions, 57 traumatic brain injuries, 55 subarachnoid hemorrhages, 32 intracranial hemorrhages, 30 subdural hematomas and 5 other indications (see Table 1). Three patients from group A and two patients from group B died in the immediate postoperative period. Mean operative time was 102.0 min (Group A) and 108.0 min (Group B) with a *p*-value of 0.24 (see Table 2; Fig. 1A). Postoperative CSF leakage was observed in 3.5% (7/201 patients, Group A) and in 3.6% (2/55 patients, Group B) with a *p*-value of 0.96 (Table 2). Postoperative shunt dependency was seen in 17.7% (35/198 patients, Group A) and in 16.4% (9/55 patients, Group B) with a p-value of 0.82 (Table 2). Postoperative hemorrhage occurred in 3.5% (7/198 patients, Group A) and 5.5% (3/55 patients, Group B) with a *p*-value of 0.52 (Table 2). Postoperative wound infections occurred in 7.1% (14/198 patients, Group A) and 1.8% (1/55 patients, Group B) with a *p*-value of 0.14 (Tables 2 and 3; Fig. 1B). Baseline demographics (age, gender, underlying pathology) were comparable between both groups.

**Table 1:** Clinical patients data

Diagnosis	No of patients
Cerebral infarction	79
TBI	57
aSDH	30
SAH	55
ICH	32
Other indications	5
Total	258

**Table 2:** Clinical and statistical results after decompressive hemicraniectomy

Clinical parameter	Group A („xenogenic dural subtitute“ group)	Group B (absent dural subtitute)	*p*-value
Mean operative time	102.0 min	108.0 min	0.24
CSF leakage	7/201 pats. (3.5%)	2/55 pats. (3.6%)	0.96
Shunt dependency	35/198 pats. (17.7%)	9/55 pats. (16.4%)	0.82
hemorrhage	7/198 pats. (3.5%)	3/55 pats. (5.5%)	0.52
wound infections	14/198 pats. (7.1%)	1/55 pats. (1.8%)	0.14

**Table 3:** View over microorganisms in cases of surgical site infection

Microorganism	Assignment to group A/B
Staphylococcus aureus	5 x in group A, 1 x MRSA in group B
Staphylococcus capitis	3 x in group A
Staphylococcus epidermidis	1 x group A
Staphylococcus agalactiae	1 x group B
Proprionibacterium acnes	1 x in group A
Enterobacter	1 x group A
E. coli	1 x group A, 1 x group B

Cranioplasty was performed in 55.8% (110/197) of the patients in Group A and in 44.4% (24/54) in Group B (*p*-value 0.14, Table 4). The use of xenogenic dural substitute did not significantly affect the duration of cranioplasty surgery (114.2 min vs. 117.6 min; *p*-value = 0.72, Table 4) or the cranioplasty results. However, the combined wound infection rate for both DHC surgery and cranioplasty showed a trend towards higher wound infection rates in patients who received xenogenic substitute in 11.6% (23/198 patients, Group A) and 3.5% (2/57 patients, Group B) with a *p-value of* 0.07 (Table 4).

**Table 4:** Clinical and statistical results after cranioplasty

Clinical parameter	Group A („xenogenic dural subtitute“ group)	Group B (absent dural subtitute)	*p*-value
Amount of cranioplasty surgery	110/197 pats. (55.8%)	24/54 pats. (44.4%)	0.14
Mean operative time	114.2 min	117.6 min	0.72
Combined wound infection rate (DHC & cranioplasty)	23/198 pats. (11.6%)	2/57 pats. (3.5%)	0.07

## Discussion

This analysis deals with the comparison of the utilization of custom made xenogenic dural substitute versus absent duraplasty. In literature there are several studies analyzing other issues of dural repair in craniotomies in general. *Azzam et al.*. reviewed the use of allografts and xenogenic grafts, where there were no significant differences in complication rates between the dural substitute groups [2]. *Güresir et al.*. examined the rapid closure technique by the onlaying of hemostyptic material onto the dural gaps, while they also did not encounter differences between their technique and the results from the literature using duraplasty [3].

*Wright et al.* analyzed the impact of single and dual-layer duraplasty during DHC, while they did not detect any significant difference between estimated blood loss and operative time with the use of a single-layer versus dual-layer duraplasty [15].

Several previous studies dealt with the size of craniectomies and craniectomy associated complications such as herniation secondary to small cranectomies, infections and cerebrospinal fluid leakages. It has been general consensus that the minimum diameter for an effective decompression should be at least 12 cm in anterior-posterior direction, with mandatory extension all the way to the base of the middle cranial fossa to minimize the risk of uncal herniation. The additional benefit of a more extensive decompression aiming for an almost holohemispherical exposure is still unclear, particularly in view of a potentially increased risk of structural laceration and other secondary complications [12].

Our study could show that the utilization of xenogenic dural substitutes did not significantly influence the prevention of postoperative CSF fistulas or the need for permanent CSF diversion after DHC. This limitations of our study is its retrospective design and single center. Statistically there was a trend towards a higher risk for postoperative infectious complications in patients who received xenogenic substitutes, which should be examined prospectively in larger multicenter studies. Biofilm formation, slower tissue integration, foreign body effect might increase infection risk with the usage of xenogenic dural substitutes. We think that the higher infectious rates in the group of patients with xenogenic dural grafts are possibly based on the foreign body reaction of the musculocutaneous and cerebral tissues to the xenogenic materials. We did not see more patients with dural substitutes suffering more severe injury including dural venous sinuses. Low number of patients were enrolled in the group B, while this should be observed and studied in a prospective fashion in order to gain certainity in the attribution of outcomes because of non-randomised design and selection bias. This analysis demonstrates that there is no difference in the postoperative complication profile with or without the usage of xenogenic dural substitutes in DHC. According to the heterogenity of the underlying pathology (TBI, malignant infarction, SAH, ICH, acute subdural hematoma) these diseases differ substantially in baseline risk for infection, CSF disturbances and surgical complexity; this issue also represents a limitation of this analysis. For practical surgery we could conclude, that the utilization of xenogenic dural substitutes for DHC did not show superiority over absent xenogenic duraplasty.

## Conclusions

The use of xenogenic substitutes may not significantly impact the prevention of postoperative complication profile with or without the usage of xenogenic dural substitutes in DHC. However, there was a trend towards a higher incidence of postoperative infectious complications in patients who received xenogenic dural substitutes.

## Data Availability

No datasets were generated or analysed during the current study.

## References

[1] Agarwalla PK, Stapleton CJ, Ogilvy CS. Craniectomy acute ischemic stroke Neurosurg. 2014;74(Suppl 1):S151–62. 10.1227/NEU.0000000000000226 24402484

[2] Azzam D, Romiyo P, Nguyen T, Sheppard JP, Alkhalid Y, Lagman C, Prashant GN, Yang I. Dural repair in cranial surgery is associated with moderate rates of complications with both autologous and nonautologous dural substitutes. World Neurosurg. 2018;113:244–248. 10.1016/j.wneu.2018.01.115. Epub 2018 Jan 31. PMID: 29374609. 10.1016/j.wneu.2018.01.115 29374609

[3] Güresir E, Vatter H, Schuss P, Oszvald A, Raabe A, Seifert V, Beck J. Rapid closure technique in decompressive craniectomy. J Neurosurg. 2011;114(4):954–60. Epub 2010 Jan 29. 20113157 10.3171/2009.12.JNS091065

[4] Heuts SG, Bruce SS, Zacharia BE, Hickman ZL, Kellner CP, Sussman ES, et al. Decompressive hemicraniectomy without clot evacuation in dominant-sided intracerebral hemorrhage with ICP crisis. Neurosurg Focus. 2013;34(5):E4. 23634923 10.3171/2013.2.FOCUS1326

[5] Inamasu J, Kaito T, Watabe T, Ganaha T, Yamada Y, Tanaka T, et al. Decom-pressive hemicraniectomy for malignant hemispheric stroke in the elderly:comparison of outcomes between individuals 61–70 and > 70 years of age. JStroke Cerebrovasc Dis. 2013;22(8):1350–4. 23489954 10.1016/j.jstrokecerebrovasdis.2013.02.008

[6] Jüttler E, Schwab S, Schmiedek P, Unterberg A, Hennerici M, Woitzik J, et al. Decompressive surgery for the treatment of malignant infarction ofthe middle cerebral artery (DESTINY): a randomized, controlled trial. Stroke. 2007;38:2518–25. 10.1161/STROKEAHA.107.485649 17690310

[7] Neugebauer H, Heuschmann PU, Juttler E. Decompressive surgery for thetreatment of malignant infarction of the middle cerebral artery—registry(DESTINY-R): design and protocols. BMC Neurol. 2012;12:115. 23031451 10.1186/1471-2377-12-115 PMC3517444

[8] Nguyen K, Reddy V, Rahimi SY. Dural sandwich technique for hemicraniectomy and benefits during cranioplasty. World Neurosurg. 2019 Apr;124:125–8. 10.1016/j.wneu.2018.12.162. Epub 2019 Jan 11. 10.1016/j.wneu.2018.12.162 30641235

[9] Park J, Hwang JH. Where are we now with decompressive hemicraniectomy for malignant middle cerebral artery infarction? J Cerebrovasc Endovasc Neu-rosurg. 2013;15(2):61–6. 10.7461/jcen.2013.15.2.61 PMC3704996 23844349

[10] Rahme R, Curry R, Kleindorfer D, Khoury JC, Ringer AJ, Kissela BM, et al. Howoften are patients with ischemic stroke eligible for decompressive hemicraniec-tomy? Stroke. 2012;43(2):550–2. 10.1161/STROKEAHA.111.635185 PMC3265663 22034001

[11] Schirmer CM, Hoit DA, Malek AM. Decompressive hemicraniectomy for the treatment of intractable intracranial hypertension after aneurysmal subarach-noid hemorrhage. Stroke. 2007;38:987–92. 17272765 10.1161/01.STR.0000257962.58269.e2

[12] Stiver SI. Complications of decompressive craniectomy for traumatic braininjury. Neurosurg Focus. 2009;26:E7. 19485720 10.3171/2009.4.FOCUS0965

[13] Suyama K, Horie N, Hayashi K, Nagata I. Nationwide survey of decompressive hemicraniectomy for malignant middle cerebral artery infarction in Japan. World Neurosurg. 2014;82(6):1158–63. 25045787 10.1016/j.wneu.2014.07.015

[14] Wagner S, Schnippering H, Aschoff A, Koziol JA, Schwab S, Steiner T. Subop-timum hemicraniectomy as a cause of additional cerebral lesions in patients with malignant infarction of the middle cerebral artery. J Neurosurg. 2001;94:693–6. 11354398 10.3171/jns.2001.94.5.0693

[15] Wright JM, Raghavan A, Wright CH, Alonso A, Momotaz H, Sweet J, Sajatovic M, Selman W. Impact of dual-layer duraplasty during hemicraniectomy on morbidity and operative metrics of cranioplasty: a retrospective case-control study comparing a single-layer with a dual-layer technique. World Neurosurg. 2019 May;125:e1189–95. 10.1016/j.wneu.2019.01.276. Epub 2019 Feb 19. 10.1016/j.wneu.2019.01.276 30794972

